# Effect of Selenium, Copper and Manganese Nanocomposites in Arabinogalactan Matrix on Potato Colonization by Phytopathogens *Clavibacter sepedonicus* and *Pectobacterium carotovorum*

**DOI:** 10.3390/plants13243496

**Published:** 2024-12-14

**Authors:** Alla I. Perfileva, Olga V. Zakharova, Irina A. Graskova, Konstantin V. Krutovsky

**Affiliations:** 1 Laboratory of Plant-Microbe Interactions, Siberian Institute of Plant Physiology and Biochemistry, Siberian Branch of the Russian Academy of Sciences, 664033 Irkutsk, Russia; alla.light@mail.ru (A.I.P.); graskova@sifibr.irk.ru (I.A.G.); 2 Scientific and Educational Center for Environmental Science and Biotechnology, Derzhavin Tambov State University, 392020 Tambov, Russia; olgazakharova1@mail.ru; 3 Department of Functional Nanosystems and High-Temperature Materials, National University of Science and Technology «MISIS», 119991 Moscow, Russia; 4 Department of Forest Genetics and Forest Tree Breeding, Faculty of Forest Sciences and Forest Ecology, Georg-August University of Göttingen, 37077 Göttingen, Germany; 5 Laboratory of Population Genetics, N.I. Vavilov Institute of General Genetics, Russian Academy of Sciences, Gubkin Str. 3, 119333 Moscow, Russia; 6 Genome Research and Education Center, Laboratory of Forest Genomics, Department of Genomics and Bioinformatics, Institute of Fundamental Biology and Biotechnology, Siberian Federal University, 660036 Krasnoyarsk, Russia; 7 Scientific and Methodological Center, G.F. Morozov Voronezh State University of Forestry and Technolgies, 8 Timiryazeva Str., 394036 Voronezh, Russia

**Keywords:** arabinogalactan, *Clavibacter sepedonicus*, colonization, copper, manganese, nanocomposite, *Pectobacterium carotovorum*, potato, selenium

## Abstract

The effect of chemically synthesized nanocomposites (NCs) of selenium (Se/AG NC), copper oxide (Cu/AG NC) and manganese hydroxide (Mn/AG NC), based on the natural polymer arabinogalactan (AG), on the processes of growth, development and colonization of potato plants in vitro was studied upon infection with the causative agent of potato blackleg—the Gram-negative bacterium *Pectobacterium carotovorum*—and the causative agent of ring rot—the Gram-positive bacterium *Clavibacter sepedonicus* (*Cms*). It was shown that the infection of potatoes with *P. carotovorum* reduced the root formation of plants and the concentration of pigments in leaf tissues. The treatment of plants with Cu/AG NC before infection with *P. carotovorum* stimulated leaf formation and increased the concentration of pigments in them. A similar effect was observed when potatoes were exposed to Mn/AG NC, and an increase in growth and root formation was also observed. The infection of plants with Cms inhibited plant growth. Treatment with each of the NCs mitigated this negative effect of the phytopathogen. At the same time, Se/AG and Mn/AG NCs promoted leaf formation. The Se/AG NC increased the biomass of *Cms*-infected plants. The treatment of plants with NCs before infection showed a decrease in the intensity of the colonization of plants by bacteria. The Se/AG NC had the maximum effect, which is probably due to its high antioxidant capacity. Thus, the NCs are able to mitigate the negative effects of bacterial phytopathogens on vegetation and the intensity of colonization by these bacteria during the infection of cultivated plants.

## 1. Introduction

Present-day climate change promotes the expansion of many phytopathogens to the new northern and eastern areas [1,2]. These microorganisms include the causative agent of potato black leg—the Gram-negative bacterium *Pectobacterium carotovorum*, which remains highly virulent at elevated temperatures, can quickly spread through the vascular system of the plant and remains latent when seeds are stored at low temperatures [3]. During the growing season, the symptoms of black leg are similar to the symptoms of potato ring rot (wilt and the yellowing of leaves), caused by the Gram-positive bacterium *Clavibacter sepedonicus* (*Cms*). Both *P. carotovorum* and *Cms* are quarantine species in many countries around the world [4,5]. Currently used pesticides are aimed primarily at combating fungal infections in potatoes and are ineffective against bacterial phytopathogens. In this regard, the development of new plant protection agents is highly needed. In order to meet this task, the possibility of using nanosubstances is being considered [6,7,8]. The use of nanocompounds for the development of agents that increase plant resistance to diseases seems promising.

Previously, we studied a number of nanocomposites (NCs) of selenium (Se) and silver (Ag) in native matrices as agents for regulating the abundance of *Cms*. The studied NCs are Se or Ag nanoparticles (NPs) stabilized by natural polymers such as arabinogalactan (AG) and isolated from Siberian larch (*Larix sibirica* L.) wood, potato starch, carrageenan and humic acids. The Se/AG NC with a Se content of 6.4% was characterized by a high antibacterial activity against *Cms* [9,10]. In addition, this NC did not have a negative effect on potato plants when visually observed over time [11,12]. However, the detailed influence of Se/AG NC on the interaction of potato with the bacterium *Cms* has not yet been elucidated. Therefore, the aim of this work is to study the influence of AG-based Se, Cu and manganese (Mn) NCs on the colonization of potato plants in vitro by bacterial pathogens *P. carotovorum* and *Cms*.

## 2. Results

**Figure 1:**
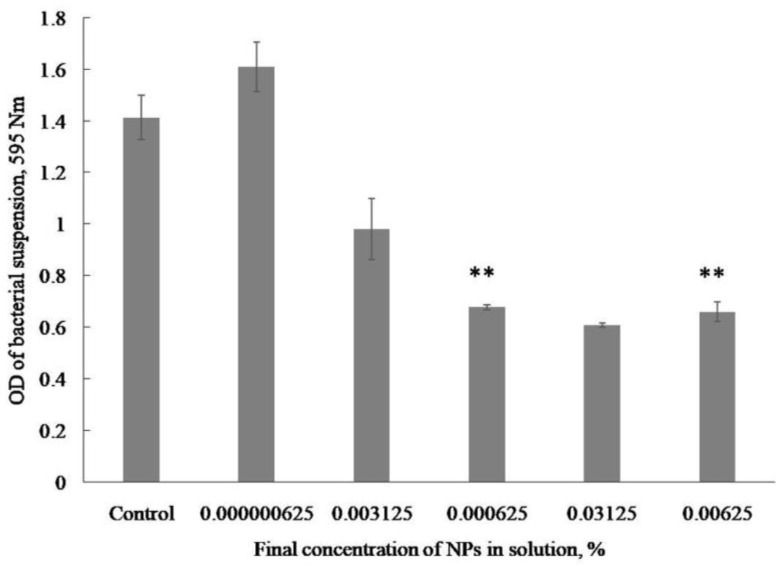
Effect of the Se/AG NC at different concentrations on the optical density (OD) of the bacterial suspension *Cms* after 24 h of incubation; ** indicates significant differences (*p* ≤ 0.01) between the control and treatments with the Se/AG NC according to the Kruskal–Wallis H-test.

The minimum inhibitory concentration (MIC) of the studied NCs was determined by the initial tests of the antibacterial effect. It was found that a high activity of the Se/AG NC, inhibiting the growth of the pathogen *Cms*, was observed at concentrations of 0.000625% and 0.00625% of Se/AG NCs in a final solution of 3 and 30 mg/mL of the Se/AG NC in an aqueous solution, respectively (Figure 1).

Thus, for further testing of the toxicity the Se/AG NC and to identify the effect of MIC concentrations of the Se/AG NC on plants, two concentrations of the Se/AG NC, 0.000625% and 0.00625%, were used in the final solution in a series of experiments. Potato plants were grown in vitro on a medium containing NCs. The condition of the plants was visually monitored during 4 days of incubation, and wilted leaves were counted. Photographs of the plants on the first day of incubation are shown in Figure S1. It was found that the first signs of leaf wilting were observed in potato plants after 2–6 h on the first day of observation in all variants with a 0.00625% concentration of the Se/AG NC (Figure S1). Potato plants treated with NCs with a Se concentration of 0.000625% remained viable at this stage of observation. On day 2, it was noted that plants treated with NCs of Se/AG at a concentration of 0.00625% had many wilted leaves, and the stems of such plants lost turgor (Figure S2). Control plants also had wilted leaves, but their number was significantly less than in all other experimental variants, except for treatment with the Se/AG NC at a concentration of 0.000625%. On day 4 of observation, the control plants remained viable. The absence of stem wilt and the yellowing of leaves was noted in the treatment with the Se/AG NC at a concentration of 0.000625%, although wilted leaves were observed. Different results were observed in the treatment with the Se/AG NC at a 0.00625% concentration, where plants died, their stems became thinner, lost turgor, and the leaves turned yellow and fell off (Figures S2 and S3).

Thus, the tests of the viability of the potato plants showed the lack of a toxic effect of the Se/AG NC at concentration of 0.000625% on potato plants during the entire observation period. Meanwhile, a 10-fold increase in the concentration of the Se/AG NC had a detrimental effect on the potatoes after 2–6 h and caused the wilting of most of the leaves and a lost of turgor in the stems at the end of the observation period.

In addition, in some of the studied plants, at the initial stage of the experiment, glutathione peroxidase (GPX) activity and the content of lipid peroxidation products (LPPs) in the tissues of potatoes treated with the Se/AG NC were studied to identify the presence of stress (Table 1).

It was found that the treatment of plants with the Se/AG NC in both concentrations did not significantly affect the activity of potato GPX, while both concentrations significantly increased the content of diene conjugates (DCs) in potato leaf tissues (Table 1).

Thus, the results of the study of phytotoxicity showed that there was a negative effect of the Se/AG NC on plants at a concentration of 0.00625%, observed 4–8 h after treatment. The presence of stress in plants was confirmed by the increased content of DCs in their tissues and a change in their GPX activity. Therefore, the Se/AG NC at a concentration of 0.000625% was used in further studies. Similarly, Mn and Cu NCs were also used at this concentration.

**Figure 2:**
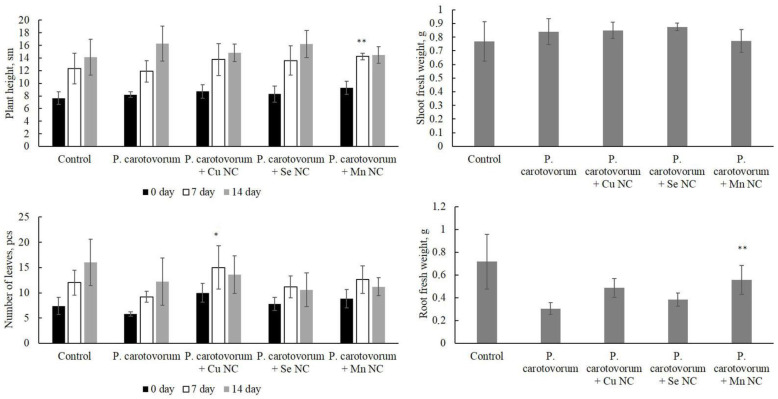
Effect of treatment with Cu/AG, Se/AG and Mn/AG NCs on potato growth (height), number of leaves, weight of fresh roots and shoots infected by *P. carotovorum*. ** *p* < 0.05 and * *p* < 0.01—significance levels when compared with infected samples without NCs treatment.

**Figure 3:**
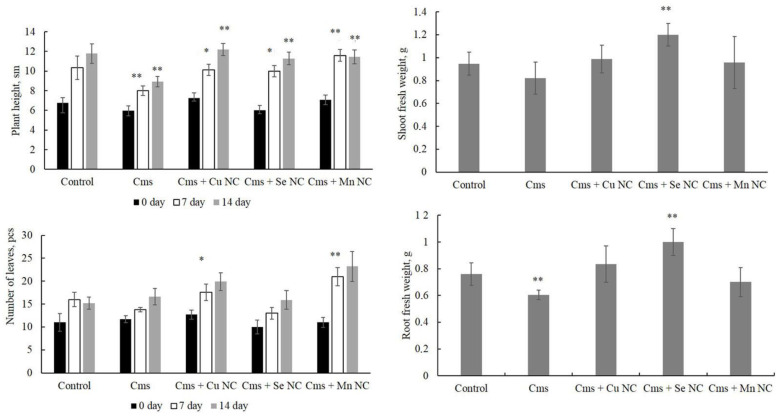
The effect of *Cms* infestation and Cu/AG, Se/AG and Mn/AG NC treatment on potato plant growth, leaf number, root weight and fresh shoot weight. ** *p* < 0.05 and * *p* < 0.01 are significance levels when compared with infected samples without the NC treatment.

The effect of phytopathogens alone and in combination with NCs on potato growth and development, as well as the biomass of roots and aboveground parts of the plants, were assessed in detail in vitro (Figure 2 and Figure 3). Plants without any treatment (pure control), as well as the effect of phytopathogens without the addition of NCs, were used as controls. In these experiments, the effect of NCs on plants alone was not studied, since such experiments have been carried out previously, and the results are described in detail in [13,14,15].

The results showed that the infection of plants with the phytopathogen *P. carotovorum* did not have a pronounced negative effect, except for the weight of roots compared to the pure control (Figure 2). The treatment of the infected potato with NCs also did not lead to any significant results, except for the apparent stimulation of potato growth (height) under the influence of the Mn/AG NC, the number of leaves under the influence of the Cu/AG NC on the 7th day of the experiment, and the weight of roots under the influence of the Mn/AG NC when compared with infected samples without NC treatment.

The results showed that the infection of plants with the phytopathogen *Cms* resulted in a decrease in the intensity of plant growth on days 7–14 of the experiment. At the same time, the treatment of plants with NCs mitigated this negative effect of *Cms* infection, all NCs stimulated plant growth on days 7 and 14 of the experiment compared to the treatment with infection only. A reliable increase in the number of leaves on day 7 of the experiment was found in plants treated with Cu/AG and Mn/AG NCs. The Se/AG NC significantly increased the biomass of the fresh shoots and the biomass of the roots of the potato plants infected with *Cms* (Figure 3).

**Figure 4:**
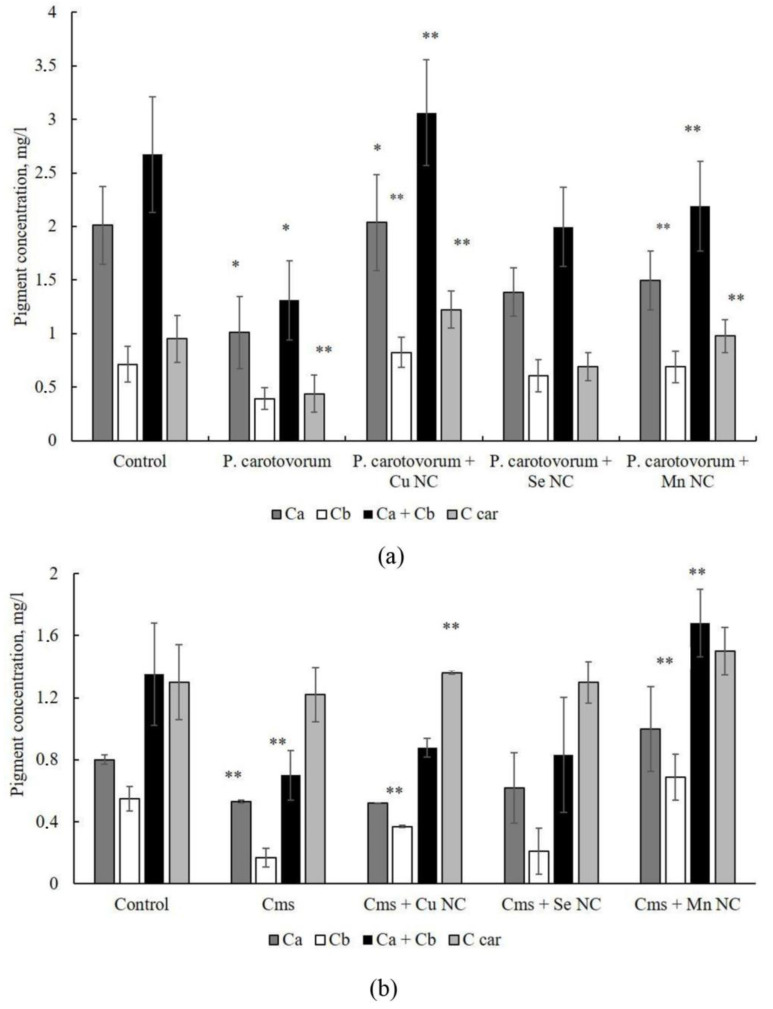
The effect of *P. carotovorum* (**a**) and *Cms* (**b**) infections and Cu/AG, Se/AG and Mn/AG NC treatment on the concentration of chlorophyll *a* (Ca) and *b* (Cb) and carotenoids (C car) in potato plant leaf tissues. ** *p* < 0.05 and * *p* < 0.01 are significance levels when compared with infected samples without NC treatment, and the *P. carotovorum* and *Cms* infections alone were compared with the non-infected control.

To assess the physiological state of the plants at the end of the experiment, the concentration of pigments in the leaf tissues was determined (Figure 4). It was found that infection with *P. carotovorum* causes a reliable decrease in the concentration of pigments in the tissues of potato leaves. The Cu/AG NC significantly increased the concentration of all the studied pigments in the tissues of infected potatoes; their values reached the levels of pigment concentration in uninfected plants. No significant stimulation of an increase in the concentration of pigments was found when treating plants with the NC Se/AG compared to infection. Mn-containing NCs increased the content of chlorophyll b and carotenoids (Figure 4a).

When infecting plants with *Cms*, a decrease in the concentration of chlorophylls was found. Cu-containing NCs increased the concentration of chlorophyll *b* in the tissues of plant leaves during infection. Se-containing NCs did not have a pronounced effect on the studied trait. Mn-containing NCs increased the concentration of chlorophylls in potato tissues during infection (Figure 4b).

The results showed that the infection of plants with *Cms* and *P. carotovorum* led to a negative effect on the morphometric parameters of plants and caused a decrease in the concentration of pigments in leaf tissues. NCs mitigated the negative effect of infection, bringing the studied parameters to the level of the control without infection. However, it is important to understand to what extent the plant was seeded with microorganisms and how NCs affect this process. To address this issue, we studied the effect of treating plants with NCs before infection on the intensity of potato colonization by *P. carotovorum* and *Cms* bacteria.

**Figure 5:**
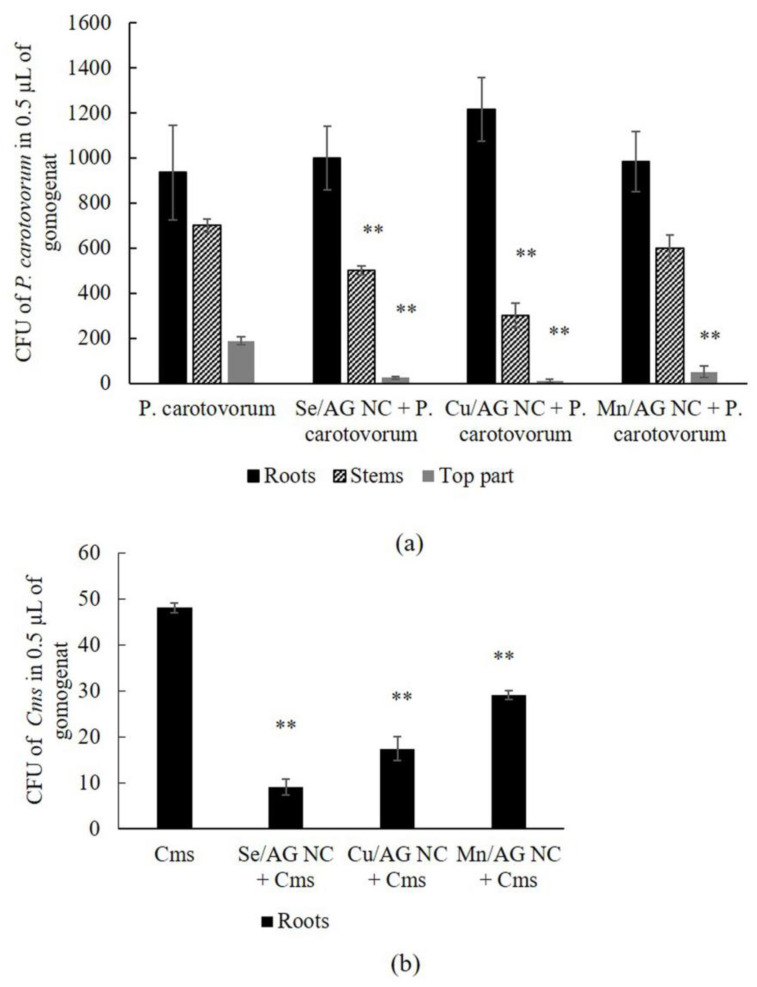
The effect of infection with *P. carotovorum* (**a**) and *Cms* (**b**) in Petri dishes on the aerial parts, stems and roots of the potato plants and treatment with Se/AG, Cu/AG and Mn/AG NCs on the intensity of the colonization of potato plants by these phytopathogens, respectively, measured as a number of colony-forming units (CFUs) of *P. carotovorum* and *Cms* in 0.5 mL homogenate. ** *p* < 0.01—significance level when compared with infected samples without NC treatment.

Potato plants were treated with NCs and incubated for 1 h, since during this time the level of ROS in potato tissues increases [10,14]. The plants were then infected with *Cms* and, after two days of coincubation and complete colonization with the pathogen, microbiological inoculations of the homogenate obtained from potato tissues were carried out according to [16]. It was found that bacteria were inoculated from all zones of the plants both in the control without NC treatment and with NC treatment (Figure 5). Please notice that, unlike (a) in Figure 5, no data are presented separately for the aerial parts, stems and roots in (b) because, unlike *P. carotovorum*, *Cms* were found only in the roots, but not in the aerial parts, leaves and stems.

A high number of CFUs of *P. carotovorum* was observed in all infected plant parts without NC treatment (Figure 5a). All of the NCs reduced bacterial CFUs in the aerial parts. The Cu/AG and Se/AG NCs reduced CFUs in the stem part of plants. The most pronounced effect was noted when treating plants with Se/AG and Cu/AG NCs (Figure 5a). The number of CFUs decreased when treating plants infected by *Cms* with any NC compared to plants untreated with NCs. The maximum effect was observed in the Se/AG NC +*Cms* treatment (Figure 5b).

## 3. Discussion

In this study, we analyzed the effect of Cu/AG, Se/AG and Mn/AG NCs embedded in an AG matrix on potatoes infected in vitro with the necrotrophic Gram-negative phytopathogenic bacterium *P. carotovorum* and the biotrophic Gram-positive phytopathogenic bacterium *Cms*. The effect of NCs on vegetation, morphometric parameters, pigment concentration and the intensity of plant colonization by the pathogen were analyzed. The use of these particular phytopathogens in our experiments was motivated by the fact that these bacteria are extremely virulent and resistant to exposure factors [17], and are quarantine objects in different countries [4,5]. Both bacteria are characterized by the ability to form biofilms, which is a key factor in the virulence of *P. carotovorum* [18,19] and *Cms*, leading to the formation of biofilms in the vascular pathways of plants and clogging them. *P. carotovorum* has a large set of pectolytic enzymes and toxins [20]. This bacterium damages plant tissues due to enzymes that destroy the plant cell wall (via pectinases, cellulases and proteinases released through type II secretion systems). The pathogenicity factors of the necrotrophic pathogen include a complex of enzymes, including pectate lyases, exopolysaccharides and lipopolysaccharides. Plants are able to demonstrate resistance due to the binding of toxins and their removal, as well as the presence of phenols that inactivate exoenzymes [21]. *Cms* also has exopolysaccharides and is able to secrete cellulase, protease, endoglucanase, xylanase, glycosyl hydrolase and serine proteases [5,19]. The defense response of potatoes to *Cms* invasion is largely associated with the activation of antioxidant enzymes during infection, but data on this response are still insufficient.

We have shown previously the fungicidal effect of the Se/AG NC against the phytopathogenic fungus *Phytophthora cactorum* [22] and the antibacterial effect of the Se/AG NC on *Cms* [13]. It was found that the Se/AG NC is able to reduce the growth of the bacterial culture of *Cms*, inhibit biofilm formation and increase the number of dead cells in the bacterial suspension [13]. In our experiments in vitro, it was revealed that the Se/AG and Mn/AG NCs have bacteriostatic, antibiofilm and bactericidal effects on *Cms* [13,14]. The antibacterial effect of NCs on *Cms* may be due to the suppression of bacterial respiration, as evidenced by a decrease in cell dehydrogenase activity and a change in the fatty acid composition of bacterial cell walls under the influence of NCs, as well as an effect on the transmembrane potential of cells [23]. There are also data on the suppression of the viability of *P. carotovorum* by nanocompounds [24]. The antibacterial activity of biosynthesized chitosan NPs against *P. carotovorum* has been demonstrated in [25]. CuS NPs inhibited the motility of *P. carotovorum* [26]. Ag NPs exhibited a high antibacterial activity against *P. carotovorum*; they completely inhibited the growth of bacteria and led to the destruction of the bacterial cell membrane and the inhibition of biofilm formation [27].

Many studies are looking at NPs as an alternative to antibiotics and other antimicrobial agents used against multi-resistant bacteria [28,29]. At the same time, there are studies in the field of medical microbiology [30] indicating that bacteria can develop resistance to metal NPs (silver, zinc and lead NPs) [31]. This is accomplished through the following mechanisms: membrane changes, reversible adaptive resistance, irreversible modifications of cell division, changes in bacterial motility and resistance, etc. One of the mechanisms of bacterial resistance to NCs is the formation of biofilms, but in our studies we monitored the effect of NCs on this process and found that NCs are still capable of destroying *Cms* [13,14] and *P. carotovorum* biofilms [24].

The surface properties, concentration and aggregation of NCs and the biofilm formation and metal exclusion ability, as well as R-plasmid and flagellin synthesis, by bacteria are decisive factors in the development of resistance to NPs in bacteria. Bacterial resistance to NPs can be avoided by modifying the surface structure of NPs or inhibiting flagellin production by bacterial pathogens. Such studies on Se NCs have not yet been conducted, but they are highly needed. However, in this article, the main focus was not so much on the antibacterial effect of NCs, but on their effect on the plant organism and its interaction with pathogenic bacteria.

The effect of nanocompounds on phytopathosystems is usually presented in the published data by studying the expression of genes and the activity of plant stress enzymes [32,33,34,35,36]. There are practically no studies on the intensity of plant colonization by the pathogen. Therefore, in our study, we studied how NCs affect the colonization of plants by the pathogen during their artificial infection and pre-treatment with an NC. The results we obtained are summarized in Table 2.

Cu and Mn belong to the group of microelements essential for living organisms. These elements play an important role in biochemical processes. Thus, in potatoes, microelements activate enzymes, participate in the synthesis of vitamins and promote the adsorption of moisture, which in turn has a positive effect on plant growth and increases their resistance to stress. Cu is a component of enzymes involved in carbohydrate and protein metabolism, and is involved in photosynthesis and respiration [37]. Cu is also involved in many vital physiological functions of plants, acting as a catalyst in oxidation-reduction reactions in mitochondria, chloroplasts and cell cytoplasm [38], or as an electron carrier in the process of plant respiration [39].

Our experiments showed that the Cu/AG NC had a positive effect on plant growth when infected with *Cms* and on their development when infected with *P. carotovorum*. The stimulating effect of the Cu/AG NC is also confirmed by published data [40,41,42,43,44,45]. For instance, it was shown that the Cu/AG NC stimulated an increase in germination and shoot and root length during the cultivation of seeds of the *Zea mays* maize hybrid variety Hema [40]. Spraying the leaves of *Dracocephalum moldavica* L. with CuO NPs increased the shoot biomass by 23% compared to the control [41]. The use of the Cu/AG NC had a positive effect on the morphological and physiological parameters of sweet basil *Ocimum basilicum* L. [42]. A positive effect of the CuO NC on the growth and development of various woody crops was also noted [43,44,45].

The stimulating effect of the Cu/AG NC can be explained by an increase in the concentration of photosynthetic pigments in the tissues of plant leaves. Cu/AG NCs are capable of influencing the intensity of photosynthesis and the functioning of the antioxidant system of plants. A number of studies confirm this [41,46]. For example, spraying the leaves of *D. moldavica* L. with CuO NCs increased the content of chlorophyll a by 77% and increased the content of chlorophyll *b* by 123% compared to the control [41]. It was shown that spraying the leaves of avocado plants with CuO NCs stimulated the intensity of photosynthesis [46].

In our experiments, the Se/AG NC did not have a pronounced effect on the growth and development of plants infected with *P. carotovorum*. However, when infecting potatoes with *Cms*, this NC stimulated all morphometric parameters: it increased the intensity of potato growth and leaf formation and root and fresh shoot biomass. Differences in the stimulating effect of this NC may be associated with its different effects on the studied pathogens. We have shown that the Se/AG NC has a complex effect on *Cms*—it suppresses biofilm formation and respiration and has bactericidal and bacteriostatic effects [10,13]. Similar effects of the Se/AG NC were also shown for *P. carotovorum*, but their expression was less pronounced, which is apparently due to the difference in the structure of the bacterial cell wall. There are published data on the positive effect of Se NPs on the physiological parameters of plants [47,48,49]. For example, exogenous spraying with nanoselenium enhances the growth of tobacco *Nicotiana tabacum* L. [47] and peanut *Arachis hypogaea* L. [48] plants. *Melissa officinalis* plants were treated with different concentrations of nanoselenium (10 and 50 mg/L) [49]. When treating plants with Se NPs at a concentration of 10 mg/L, a sharp increase in biomass, an activation of lateral buds and the stimulation of lateral root development were observed [49].

In the experiments presented in this paper, a high biological activity was observed for the Mn/AG NC. This activity of the Mn/AG NC may be associated with the small size of Mn NPs. It is known that the absorption of Mn NPs by plants depends on their size. Typically, the pore size of plant cell walls is in the range of 3–8 nm, so large Mn NPs will not penetrate cell walls [50], and Mn NPs in the NCs we studied were in the specified size range of 3–6 nm [14]. The Mn/AG NC stimulated plant growth and development during infection in both experimental variants, and also stimulated root formation during the infection of plants with *P. carotovorum*. The stimulating effect of Mn is described in the published data [51,52,53,54]. For example, it was shown that MnFe_2_O_4_ NPs were captured from the roots and migrate to the leaves, which helps to improve the growth parameters of barley [51]. FeO_x_ NPs, MnO_x_ NPs and bimetallic MnO_x_/FeO_x_ NPs had positive effects on the growth of *Zea mays* plants, particularly on the seed germination rate, root growth and biomass growth of maize seedlings [52]. MnO_2_ NPs increased plant length, root length and leaf and flower numbers in bean (*Phaseolus vulgaris*) [53]. The application of ZnO-MnO NPs at a concentration of 270 ppm increased root size, dry biomass, chlorophyll content and leaf area in cabbage (*Brássica oleracea*) plants [54].

The stimulating effect of NC Mn/AG may be associated with an increase in the concentration of pigments in potato leaf tissues under its influence. Mn is known to affect the photosynthetic activity of plants [53,55,56]. This effect is associated with increased chloroplast stability, enhanced chlorophyll and carotenoid biosynthesis, increased ROS inactivation, the activation of H^+^-ATPase gene expression, the activation of ribulose-1,5-bisphosphate carboxylase/oxygenase (Rubisco), the improved absorption of mineral elements, effects on the electron transport chain and oxidative phosphorylation and effects on ATP and NADPH synthesis. It is believed that the mechanism of activation of photosynthesis under the influence of metal oxide NPs is associated, due to their small size, with their ability to penetrate plant chloroplasts, reach the reaction center of photosystem II and enhance electron transfer and light absorption in plant chloroplasts [57].

In the present study, a decrease in the intensity of plant root colonization by *P. carotovorum* and *Cms* was revealed. At the same time, the NCs did not completely block the penetration of bacteria into plant roots. This is probably due to the high infectious load on plants under the conditions of the model experiment and the fact that roots are the first plant organ to come into contact with pathogens. In addition, NC treatment occurred before plant infection. As a result, NPs from the NC composition could already move with the xylem flow to the aboveground plant organs, where the effect of some NCs has already been noted. It has been shown that roots absorb NPs through the main root, pores in the root cell wall and damaged areas [58]. The intensity of colonization of the stem zone of plants decreased under the influence of the Cu/AG NC when plants were infected with *P. carotovorum* and under the influence of the Se/AG NC when potatoes were infected with *Cms*. A decrease in the contamination of the apical zone of potatoes was observed under the influence of all NCs. The decrease in the intensity of plant colonization by *P. carotovorum* in the apical zone of plants may be due to the fact that the pre-treatment of plants with NCs led to the absorption of NCs by the plant and its distribution throughout the plant, which could lead to an increase in the activity of antioxidant enzymes and an effect on the expression of protective genes. It is known that Se NPs have high antioxidant activity [59]. Thus, it is known that Se NPs affect the activity of antioxidant enzymes in various plant organs—nitrate reductase in leaves and PER in roots [34]. It has been shown that metal NPs can affect the expression levels of genes encoding vacuolar proton exchanger synthesis, superoxide dismutase (SOD), cytochrome P450-dependent oxidase and peroxidase [60], microRNA genes [61] and genes encoding aquaporins in seeds [62]. Takehara et al. [63] demonstrated that MgO NPs induce strong immunity against *Fusarium* wilt in tomato, which is also associated with differential expression of several genes. These data provided new insights into the possible genetic mechanisms explaining this immunity. It is known that gene expression in plant cells can also be altered by Cu NP exposure. Thus, it was shown on the downy birch *Betula pubescens* that the infection of plants with the pathogen *Alternaria alternata* significantly increased the level of transcripts of the MYB46 transcription factor, LEA8 protective proteins, phenylalanine-ammonia lyase PAL and pathogen-dependent proteins PR-1 and PR-10 in birch microclones. When NPs were added to the cultivation medium and simultaneously exposed to the phytopathogen, the expression of the *MYB46*, *PR-1* and *PR-10* genes decreased by 5.4 times. The obtained effect is associated with a decrease in the pathogenic load caused by exposure to NPs and simultaneous stimulation of clones in vitro [45]. In the tissues of *Glycine max* soybean seedlings treated with CuO NPs, a decrease in the expression activity of a number of genes involved in the cell division process was found [64]. It has been shown that the use of chitosan–polyvinyl alcohol hydrogel (Cs-PVA) in combination with Cu NPs promotes an increase in the expression of genes encoding the synthesis of jasmonic acid and SOD in tomato plant tissues under salt stress, mitigating its consequences [65]. A decrease in the expression of the pathogen-associated protein 1 (PR1) and polyphenol oxidase precursor (PoP) genes was observed in the tissues of *Capsicum annuum* pepper and tomato when treating plants infected with the pathogen *Xanthomonas euvesicatoria* with an NC consisting of Cu NPs and Ag NPs that includes Ag deposited on reduced graphene oxide [66].

For example, it was shown that the foliar application of Cu NPs improved the quality of *Solanum lycopersicum* tomato fruits and increased the synthesis of biologically active compounds, as well as the antioxidant effects of catalase (CAT) and SOD [67]. It was shown that spraying maize plants with Cu NPs in combination with aspartic acid under field experiment conditions leads to a significant decrease in ROS in the treated plants due to an increase in the activity of antioxidant enzymes (peroxidase, SOD, ascorbate peroxidase, etc.) under the stress caused by lead toxicity [68]. It was shown that the treatment of *Phaséolus vulgáris* bean plants with Cu NPs reduced ROS generation by increasing the activity of antioxidant enzymes (CAT, ascorbate peroxidase, etc.), as well as inhibiting the activity of ROS-producing enzymes, such as GPX and NADPH oxidase. In addition, under the influence of Cu NPs, the amount of malondialdehyde in bean tissues decreased by 50% [69]. Some NPs, such as Mn nanoforms, exhibit antioxidant activity against ROS [70,71]. The general mechanism of the antioxidant effect of Mn NPs is associated with the induction of photosynthesis processes, the stimulation of SOD activity, and an increase in the level of proline and phenolic compounds [72]. The antioxidant effect of Mn NPs is explained by the high content of Mn ions in the NPs, which are capable of capturing free radicals and neutralizing them. Therefore, Mn NPs, unlike other antioxidants, such as ascorbic acid, have a prolonged effect. MnO_2_ NPs affect the activity of antioxidant enzymes: CAT, SOD and GPX. They inactivate ^•^OH and inhibit apoptosis processes induced by a high content of ROS in animal cell tissues [73]. Due to such antioxidant properties, Mn NPs have been actively studied for medicinal purposes, in particular for their anticancer activity [74]. Mn_3_O_4_, MnO and MnO_2_ have enzyme-like activities, which allow them to inactivate ROS [75,76,77]. The stimulation of SOD by 28.16%, ascorbate peroxidase by 52.38% and CAT by 28.57% was observed in rice seeds nanoprimed by FeS and MnS NPs in comparison with the control (seeds primed only by water) [78]. Mn_3_O_4_ NPs have enzymatic activity similar to SOD and CAT, and were also capable of scavenging hydroxyl radicals [77].

Thus, the published results and the data obtained in this paper confirm that treatment with Mn, Cu and Se NPs increases plant resistance to pathogen infection. The maximum effect in limiting bacterial penetration was found for the Se/AG NC, which can be explained by the high antioxidant capacity of the Se/AG NC compared to metal-containing NC.

## 4. Materials and Methods

### 4.1. Synthesis of Nanocomposites (NCs)

For the synthesis of NCs, AG was obtained from the polysaccharide of Siberian larch, *Larix sibirica* Ledeb. (OOO Wood Chemistry, Irkutsk, Russia). It was additionally purified to remove impurities and flavonoids by passing it through a polyamide column. To synthesize a Se-containing NC, 1 g of AG polysaccharide and 0.136 g of sodium bis(2-phenylethyl) diselenophosphinate were placed in a reaction flask, then, 50 mL of water were added and the mixture was stirred with a magnetic stirrer until the reagents were completely dissolved. The solution was additionally thermostated for 3 h at 35–40 °C, and concentrated (30%) hydrogen peroxide was added. The Se/AG NC was isolated and purified from the resulting sodium diphenylphosphinate by pouring the reaction mixture into a fourfold excess of acetone or ethanol, followed by washing on a filter with the same solvent, filtering the precipitate and drying in air. The yield of the NC with a Se content of 3.4% was 97% (in terms of Se from its precursor). The obtained Se/AG NC is a water-soluble orange-red powder, well soluble in water. X-ray phase analysis using a Bruker D8 ADVANCE diffractometer (Bruker Corporation, Billerica, MA, USA) showed an X-ray amorphous structure of the Se/AG NC. The formation of red amorphous Se NPs was identified by the appearance of intense absorption in the visible region of the spectrum (310 nm). The absence of any reflections on the X-ray diffraction pattern of the Se/AG NC also indicates that the known X-ray amorphous allotropic modification of elemental Se is performed. Using a transmission electron microscope (TEM), it was revealed that the Se NPs are easily visualized and have a shape that is close to spherical. The size of the NPs in the Se/AG NC was 20–65 nm, with an average value of 25 nm. Se nanoparticles are fairly uniformly distributed in the polysaccharide matrix [13].

Mn/AG NCs were synthesized by first dissolving AG (2 g, 18 kDa) in H_2_O (5 mL) and adding MnSO_4_ × 5H_2_O (0.4 g) in H_2_O (3 mL) to the solution. Then, NH_4_OH (0.1 mL) and hydrazine (0.2 mL) were added under magnetic stirring. After stirring for 3 h, the reaction mixture was precipitated with alcohol and dried. Washing with alcohol yielded 1.68 g of Mn/AG NC. The Mn NPs were most likely formed as Mn(OH)_2_ × nH_2_O and were stabilized in the matrix on oxygen atoms and hydroxyl groups. According to the TEM data, electron-dense round Mn NPs with an average size of 3–6 nm were generated in the films obtained by NCs in the matrix. NPs hardly formed cluster groups and were usually located at a distance from each other in the polymer volume. The Mn/AG NCs are paramagnetic [14,79].

The Cu-containing NC was synthesized by adding 2 mL of an aqueous solution of CuCl_2_ containing 0.09 g CuCl_2_•2H_2_O to a solution of 1 g of AG in 6 mL of water with vigorous stirring. The mixture was kept at 320 K for 30 min; then, 5 mL of an aqueous solution containing 0.08 g of NaBH_4_ and 0.003 g of NaOH were added; the reaction mixture was kept for 3 h with vigorous stirring and filtered through a paper filter. The target product was isolated from the filtrate and purified from low-molecular impurities by double precipitation from ethyl alcohol, and dried in a vacuum. The Cu content in the NC sample, determined by X-ray energy-dispersive microanalysis, was 7.5%. According to the TEM, the sizes of the Cu (I) oxide NPs in the Cu/AG NCs were in the range of 1–26 nm, with predominant particles of 2–5 nm [15].

### 4.2. Potato Plants

Potato plants of the Lukyanovsky variety, susceptible to infections, were used in the experiments in vitro [80]. The microclonal propagation of the plants was carried out in test tubes using cuttings on Murashige–Skoog agar nutrient medium (Sigma-Aldrich, Saint Louis, MO, USA). The plants were cultivated under controlled conditions for 20 days at 26 °C and under illumination at 5–6 klx.

### 4.3. Bacteria

The strains of the potato ring rot pathogen *Cms* Ac-1405 and phytopathogenic Gram-negative bacterium *Pectobacterium carotovorum* subsp. *carotovorum* strain VKM B-1274 were obtained from the All-Russian Collection of Microorganisms (G.K. Skryabin Institute of Biochemistry and Physiology of Microorganisms, Russian Academy of Sciences, Pushchino, Moscow Region, Russia). *Cms* bacteria were grown on a medium with glucose, peptone and yeast extract (GPY) [81]. *P. carotovorum* bacteria were cultured on meat peptone (MP) agar media (State Research Center for Applied Microbiology and Biotechnology, Obolensk, Moscow Region, Russia); for the experiments, they were grown on MP broth. The antibiofilm effect of NCs was determined using the plate method [82]. The minimum inhibitory concentration (MIC) of the Se/AG NC in relation to *Cms* was studied using the broth dilution method [83]. The optical density of the *Cms* bacterial suspension after 24 h of incubation with different concentrations of Se/AG NC (5 concentrations) was recorded at 595 nm on a plate spectrophotometer, model 680 (Bio-Rad Laboratories, Inc., Hercules, CA, USA).

### 4.4. Study of Phytotoxicity of Nanocomposites (NCs)

To study the phytotoxicity of the Se/AG NCs, potato plants of the Lukyanovsky variety were placed in vitro in containers with a cultivation medium (liquid nutrient medium Murashige–Skoog). Control untreated plants and plants treated with the Se/AG NC by their incubation in a medium with the addition of the Se/AG NC at a concentration of either 0.000625% or 0.00625% were used in the experiment.

During the first day, the number of wilted leaves was recorded every 2 h (at 0, 2, 4, 6 and 8 h). The experiment continued for 4 days. Some of the plants from this experiment were used to collect samples on the 1st day of the experiment to measure biochemical parameters—GPX activity (after 3 h of incubation with the NC) and the content of DC (after 24 h of incubation with the NC) in the tissues of the potato leaves. The determination of the primary products of LPO and DC in the tissues of the potato plants was carried out according to the standard method using hexane and isopropanol [84]. The GPX activity in the potato tissues was determined according to the Boyarkin method [85].

### 4.5. Nanocomposite Treatment

In a series of experiments on the effect of phytopathogen infection and NC treatment on the potato growth rate, potato plants at the age of 4 tillering nodes were transferred to a liquid Murashige–Skoog nutrient medium in test tubes with the addition of an aqueous solution of Se/AG, Mn/AG and Cu/AG NCs separately, one plant per test tube (a total of 5 test tubes per variant). After 1 h of incubation of the plants with NC, 1 mL of a bacterial suspension of the phytopathogen (titer 4 × 10^9^ colony forming units (CFUs) per ml) per 10 mL of medium was added to the Murashige–Skoog nutrient medium in the test tubes. In the present experiments, variants with only the NC treatment of plants without infection were not included due to the fact that such studies were conducted in previous works and the absence of a negative effect of NC on potato plants was shown [13,14,15]. Plants for measuring biometric parameters and for the experiment on the effect of NC on the intensity of colonization by phytopathogens were prepared simultaneously (see Table 3).

In a series of experiments on the effect of infection and NC on the biometric characteristics of potato plants, the plant growth rate and leaf count were recorded daily for 7 days. At the end of the experiment, the mass of fresh shoots and roots, and the pigment content in leaf tissues, were measured in the experiment with the phytopathogen and in the control without it. The pigment content was determined spectrometrically using 80% acetone [86].

The intensity of the colonization of potato plants by bacteria was determined using microbiological cultures. After incubation for two days together with a bacterial suspension of the phytopathogen under controlled conditions, the plants were sterilized for 10 min in a solution of 10% sodium hypochlorite with the addition of two drops of Tween-80 detergent (Sigma-Aldrich, Saint Louis, MO, USA) and washed three times with sterile water. The roots, stems and apical parts of plants were then separately ground in a sterile porcelain mortar and pestle. The resulting homogenate was diluted multiple times and plated on YPGA medium by grinding it into a dish with a Drygalski spatula. The dishes were incubated at 26 °C in the dark for 7 days, and then CFUs were determined.

The obtained data were statistically analyzed using the Microsoft Excel software package and the SigmaPlot v.12.5 program (SYSTAT Software, Chicago, IL, USA). The Shapiro–Wilk test was used to check the measurements for normality. The data obtained after treatment were statistically compared with controls using either the nonparametric Kruskal–Wallis U-test for traits that did not follow normal distribution or Student’s test for traits that followed normal distribution.

## 5. Conclusions

It was shown in this study that the infection of potatoes with phytopathogenic bacteria leads to a decrease in the morphometric parameters of potatoes in vitro, a decrease in the concentration of pigments in leaf tissues, and an intensive colonization of all plant zones by the pathogen *P. carotovorum* and the root zone by the pathogen *Cms*. The treatment of plants with NCs mitigated these negative effects of the phytopathogens. The treatment of plants with the Cu/AG NC before infection with *P. carotovorum* stimulated leaf formation and increased the concentration of pigments in them. A similar effect was observed when potatoes were exposed to the Mn/AG NC, and an increase in growth and root formation was also observed. Both the Se/AG and Mn/AG NCs promoted leaf formation. The Se/AG NC also increased the biomass of *Cms*-infected plants. The treatment of plants with each NC before infection showed a decrease in the intensity of the colonization of the apical zones of plants by the bacteria. The Se/AG NC had the maximum effect, which is probably due to its high antioxidant capacity. The presented data show the potential of the studied NCs for use as phytoprotectors for valuable agricultural plants.

## Figures and Tables

**Table 1:** Effect of Se/AG NC at two concentrations, 0.000625% and 0.00625%, on glutathione peroxidase (GPX) activity and diene conjugate (DC) content in potato leaf tissues in vitro.

Treatment	GPX, Relative Units	DC (Nm/g Wet Weight) × 10^6^
Control	0.7836 ± 0.1189	0.5513 ± 0.0463
Se/AG NC (0.000625%)	0.8119 ± 0.0269	0.7871 ± 0.0689 *
Se/AG NC (0.00625%)	0.9335 ± 0.1007	0.7031 ± 0.0899 *

* *p* ≤ 0.01 indicates significant differences between the control and treatments with the Se/AG NC according to the Kruskal–Wallis H-test.

**Table 2:** Summary data on the effect of the infection of potato plants with phytopathogens *P. carotovorum* and *Cms* and the effect of their treatment with the Cu/AG, Se/AG and Mn/AG NC on the morphometric parameters, pigment concentration and bacterial contamination intensity.

Treatment	*P. carotovorum*	*Cms*
Infection	reduction in root biomass	decrease in plant growth rate
	reduction in pigment concentration	decrease in pigment concentration
Cu/AG NC + Infection	stimulation of leaf formation during infection	stimulation of plant growth intensity
	increase in pigment concentration	increase in chlorophyll b concentration
	reduction in CFUs of the phytopathogen in the apical and stem zones of plants	reduction in CFU of phytopathogen in the root zone of plants
Se/AG NC + Infection	reduction in CFUs of phytopathogen in the apical and stem zones of plants	reduction in pathogen phytotoxicity
		stimulation of plant growth intensity and leaf formation
		increase in biomass of fresh shoots and roots
		reduction in CFU of phytopathogen in the root zones of plants
Mn/AG NC + Infection	reduction in pathogen phytotoxicity	stimulation of plant growth rate and leaf formation
	stimulation of plant growth and leaf formation during infection	increase in chlorophyll concentration
	increase in root formation	
	increase in the concentration of chlorophyll b and carotenoids	reduction in CFU of phytopathogen in the root zones of plants
	reduction in CFUs of the phytopathogen in the apical zones of plants	

**Table 3:** The plant material used in the treatment variants and control.

Treatment Variant	Number of Plants Used for Measuring	
	Biometric Parameters Taken Daily for 7 Days	Intensity of Colonization by the Pathogen Seeded Two Days After Infection with the Phytopathogen
Control (without infection and NC)	5	3
*P. carotovorum*	5	3
*P. carotovorum* + Se/AG NC	5	3
*P. carotovorum* + Mn/AG NC	5	3
*P. carotovorum* + Cu/AG NC	5	3
*Cms*	5	3
*Cms* + Se/AG NC	5	3
*Cms* + Mn/AG NC	5	3
*Cms* + Cu/AG NC	5	3

## Data Availability

Data are contained within the article.

## Supplementary Materials

The following supporting information can be downloaded at: https://www.mdpi.com/article/10.3390/plants13243496/s1, Figure S1: The evaluation of the viability of potato plants in the first 0, 2, 4, 6 and 8 h on the first day of observation after treatment with a solution of the Se/AG NC at concentrations of 0.000625% and 0.00625%. C—control; Figure S2: The evaluation of the viability of potato plants on the 2nd and 4th days of observation after treatment with a Se/AG NC solution at concentrations of 0.000625% and 0.00625%; Figure S3: The effect of the Se/AG NC at concentrations of 0.000625% and 0.00625% on the number of wilted potato leaves in vitro.
