# Supplementary material for: Effect of Selenium, Copper and Manganese Nanocomposites in Arabinogalactan Matrix on Potato Colonization by Phytopathogens Clavibacter sepedonicus and Pectobacterium carotovorum
Source: Plants (Basel). 2024 Dec 14;13(24):3496. doi: 10.3390/plants13243496 (PMC11677604; doi:10.3390/plants13243496)
Supplement: Supplementary file 1 [file plants-13-03496-s001.zip › plants-3353168-supplementary.pdf]

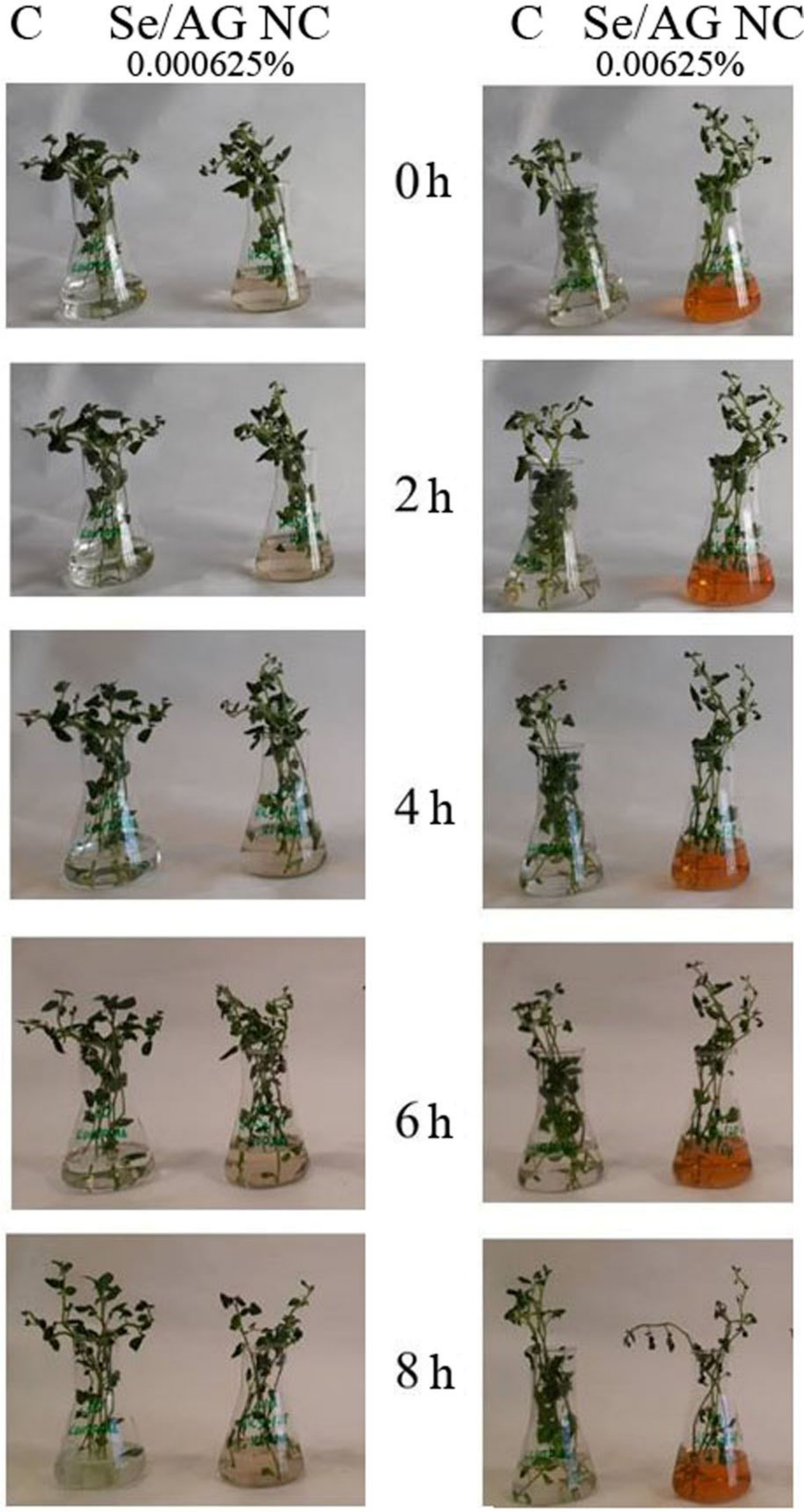

Figure S1. Evaluation of the viability of potato plants in the first 0, 2, 4, 6 and 8 hours on the first day of observation after treatment with a solution of Se/AG NC at concentrations of 0.000625% and 0.00625%. C – control.

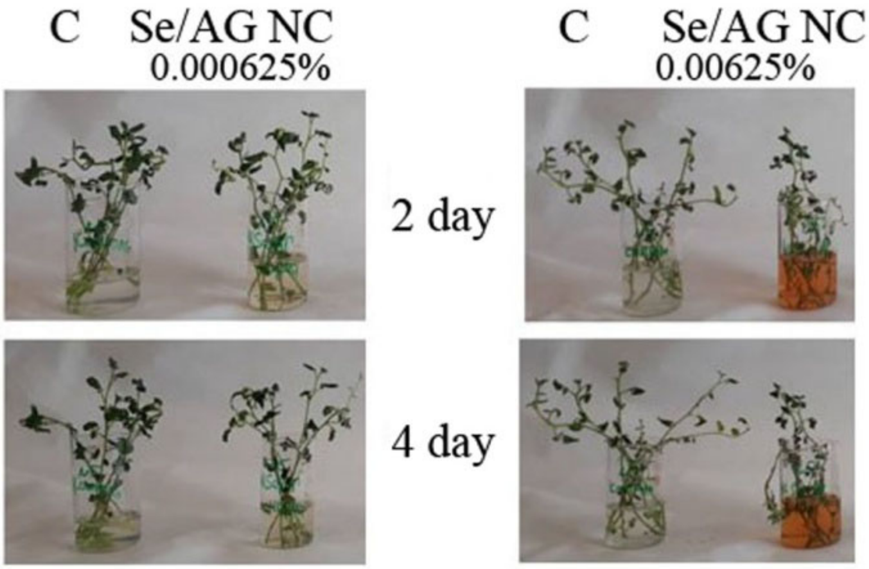

Figure S2. Evaluation of the viability of potato plants on the 2nd and 4th days of observation after treatment with a Se/AG NC solution at concentrations of 0.000625% and 0.00625%.

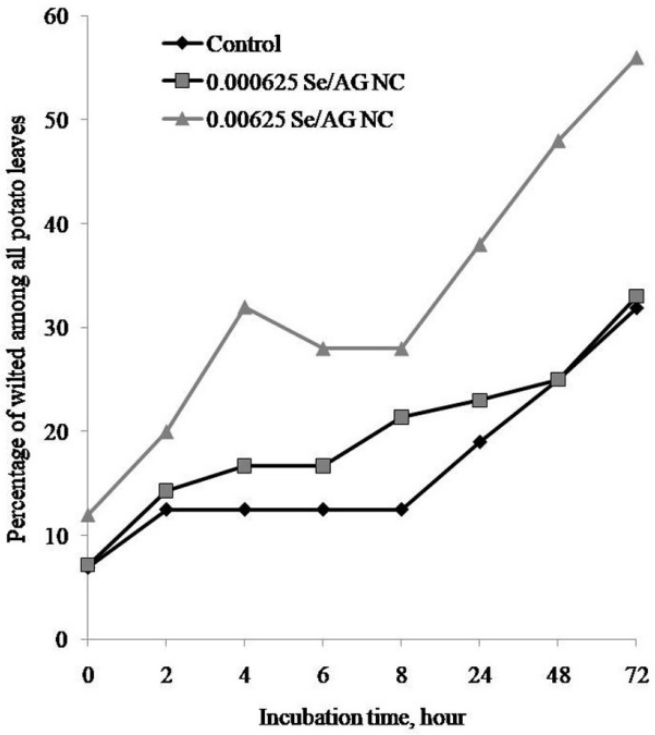

Figure S3. Effect of Se/AG NC at concentrations of 0.000625 % and 0.00625 % on the number of wilted potato leaves *in vitro*.
